# Plant-induced bacterial gene silencing: a novel control method for bacterial wilt disease

**DOI:** 10.3389/fpls.2024.1411837

**Published:** 2024-08-02

**Authors:** Seonghan Jang, Doyeon Kim, Soohyun Lee, Choong-Min Ryu

**Affiliations:** ^1^ Infectious Disease Research Center, KRIBB, Daejeon, Republic of Korea; ^2^ Department of Biosystems and Bioengineering Program, Korea Research Institute of Bioscience and Biotechnology (KRIBB) School of Biotechnology, University of Science and Technology (UST), Daejeon, Republic of Korea; ^3^ Department of Pediatrics, University of California at San Diego, La Jolla, CA, United States

**Keywords:** host-induced gene silencing, plant-induced bacterial gene silencing, *Ralstonia pseudosolanacearum*, *Agrobacterium tumefaciens*, *Tobacco rattle virus*

## Abstract

*Ralstonia pseudosolanacearum*, a notorious phytopathogen, is responsible for causing bacterial wilt, leading to significant economic losses globally in many crops within the Solanaceae family. Despite various cultural and chemical control strategies, managing bacterial wilt remains a substantial challenge. This study demonstrates, for the first time, the effective use of plant-induced bacterial gene silencing against *R. pseudosolanacearum*, facilitated by *Tobacco rattle virus*-mediated gene silencing, to control bacterial wilt symptoms in *Nicotiana benthamiana*. The methodology described in this study could be utilized to identify novel phytobacterial virulence factors through both forward and reverse genetic approaches. To validate plant-induced gene silencing, small RNA fractions extracted from plant exudates were employed to silence bacterial gene expression, as indicated by the reduction in the expression of GFP and virulence genes in *R. pseudosolanacearum*. Furthermore, treatment of human and plant pathogenic Gram-negative and Gram-positive bacteria with plant-generated small RNAs resulted in the silencing of target genes within 48 hours. Taken together, the results suggest that this technology could be applied under field conditions, offering precise, gene-based control of target bacterial pathogens while preserving the indigenous microbiota.

## 1 Introduction

The soil-borne bacterial pathogens*, Ralstonia solanacearum* and *R. pseudosolanacearum*, are causative agents of bacterial wilt, which has a significant economic impact on many plant species including those of the Solanaceae family (Genin and Denny, 2012). Although various cultural control methods have been developed, effective disease management remains elusive. Plant breeding bacterial wilt-resistant lines is effective but requires time-consuming and labor-intensive processes. Resistance breaking has also been observed when plants are cultivated continuously in the field (Koch and Wassenegger, 2021). Thus, antibiotics have been employed to inhibit pathogen growth more effectively. However, prolonged use has led to the emergence of antibiotic-resistant *Ralstonia* spp., causing unintended consequences such as negative impacts on indigenous microbiota and potential risks to animal and human health (Sundin and Wang, 2018). Therefore, novel methods to control these pathogens are urgently needed.

Besides bacterial disease control, host-induced gene silencing (HIGS) has been developed to control eukaryotic pathogens and pests using vector constructs carrying DNA fragments from phytopathogenic fungi, insects, or nematodes, rather than plant genes (Nowara et al., 2010). Prior to the stable transformation of the hairpin-structure-based RNAi construct, scientists evaluated the potential of HIGS by conducting tests on virus-induced gene silencing (VIGS). VIGS employs engineered plant virus vectors that are used either to directly inoculate host plants or infect them through *Agrobacterium tumefaciens* strains, which introduce the viral genome into plant cells (Burch-Smith et al., 2004). Once plant cells are infected by the viral vectors carrying transgene constructs, transcription and replication occur, producing sequence-specific double-stranded RNA (dsRNA). The dsRNA is then processed by the plant’s RNAi machinery into small interfering RNAs (siRNAs). The siRNAs are recruited into the RNA-induced silencing complex (RISC), suppressing target mRNAs (Hammond et al., 2001). *Tobacco rattle virus* (TRV) is widely used as a VIGS vector due to its mild disease symptoms, high efficiency of gene silencing, and broad host range (Ratcliff et al., 2001). As TRV is a bipartite virus, the TRV-based VIGS system utilizes two vectors: pTRV1 and pTRV2 (**Supplementary Figure S1**). pTRV1 encodes the elements necessary for viral replication and movement, allowing it to establish a systemic infection throughout the plant. pTRV2 can be modified to contain a fragment of the target gene to induce RNA silencing. Inoculating *Nicotiana benthamiana* plants with *A. tumefaciens* strains carrying pTRV1 and pTRV2 effectively achieves VIGS of the target gene (Robertson, 2004).

The goal of the TRV-mediated HIGS or VIGS systems is the control of eukaryotic agricultural pests and pathogens. In this system, pTRV1 and engineered pTRV2 vectors collaboratively synthesize infectious TRV particles within transfected plant cells. Upon systemic spread, the replicating virus produces siRNAs targeting vital genes in invading eukaryotic pathogens. The generated siRNAs can move cell-to-cell via the plasmodesma and phloem, and they can also be transported to the eukaryotic pathogens when the plants are under pathogen attack. The transferred pathogen-specific siRNAs trigger RNAi, attenuating the disease and reducing pest infestation. Recent studies also indicate that plants can secrete siRNAs via extracellular vesicles (EVs), which are nanoscale particles released from plant cells enclosed by a lipid bilayer, or non-extracellular vesicles (non-EVs), although typically a small proportion of exported siRNAs are enclosed in EVs (Cai et al., 2018b).

However, the application of these techniques for the control of prokaryotic phytopathogens is limited because bacteria lack an RNAi machinery or small RNA (sRNA) delivery system analogous to that of eukaryotes (Singla-Rastogi et al., 2019). Nonetheless, there is evidence that eukaryote-derived sRNAs can regulate bacterial gene expression (Liu et al., 2016). In mammals, microRNAs (miRNAs) enclosed in intestinal EVs have been shown to influence the composition of the bacterial gut microbiota by entering the bacteria and regulating gene transcription (Liu et al., 2016). Similarly, exosome-like nanoparticles from ingested edible plants deliver sRNAs and miRNAs to gut bacteria, modulating the gut microbiota and their metabolites (Teng et al., 2018). Even in plants, plant-derived miRNAs have been shown to be delivered to bacteria. For example, *Arabidopsis thaliana* and *Brachypodium distachyon* secrete miRNAs from their roots, which are subsequently absorbed by the rhizosphere microbiome and even soil bacteria located far from the roots (Middleton et al., 2024). Considering that TRV leaf-infiltration effectively silences genes in the root of *N. benthamiana* (Ryu et al., 2004), it is plausible that siRNAs or miRNAs produced in plants could silence virulence genes in root-associated prokaryotic phytopathogens. However, no studies have yet shown that plant-induced sRNAs silence genes in bacterial pathogens to attenuate their virulence.

In this study, the objective is to provide a proof-of-concept of the effectiveness of plant-induced bacterial gene silencing (PIBGS) based on the HIGS system. To achieve this objective, we targeted well-known virulence genes as well as random genes in *R. pseudosolanacearum* strain SL1931, which shows hyper virulence toward *N. benthamiana* (Riu et al., 2022), by using both forward and reverse genetic approaches to validate the efficiency of PIBGS for controlling the root-associated prokaryotic phytopathogen.

## 2 Materials and methods

### 2.1 Plant materials and growth conditions

Seeds of *N. benthamiana* were surface-sterilized with 6% sodium hypochlorite, rinsed three times with sterilized distilled water, and subsequently sown in a sterile, soil-less potting medium (Punong Co. Ltd., Gyeongju, South Korea). *N. benthamiana* plants were then cultivated at 25 ± 2°C, under a cycle of 12 h of light and 12 h of dark, with a light intensity of approximately 7,000 lux from fluorescence lamps. At 10 days old, *N. benthamiana* seedlings were transplanted into plastic pots with a diameter of 9 cm to continue their growth.

### 2.2 Gene cloning

#### 2.2.1 Virulence factors

Genomic DNA of *R. pseudosolanacearum* was extracted using the Wizard Genomic DNA purification kit (Promega, Madison, WI, USA) following the manufacturer’s instructions. Partial gene fragments related to virulence factors (**Supplementary Table S1**) in *R. pseudosolanacearum* were amplified through diagnostic PCR using specific primers that include sequences overlapping with those of the pTRV2 vector at their 5’-ends (**Supplementary Table S2**). For cloning into the pTRV2 vector, the vector was digested with the SmaI restriction enzyme and assembled with amplified DNA fragments using the Cold Fusion Cloning Kit (System Biosciences, California, USA). Cloned vectors (1 ng) were then transformed into *Escherichia coli* DH5α competent cells via heat shock at 42°C for 45 secs. The transformed *E. coli* cells were plated on Luria-Bertani (LB) agar plates containing 100 μg/mL kanamycin and incubated at 37°C for 24 h. Colonies with the cloned pTRV2 vector were identified through diagnostic PCR using pTRV2-F (5’-TTACTCAAGGAAGCACGATGA-3’) and pTRV2-R (5’-CCTACGAGATTGACATTCTC-3’) as primers. The inserted gene sequences were verified by sequencing and pTRV2 vectors with the transgene were then transformed into *A. tumefaciens* GV2260 electrocompetent cells using the Gene Pulser Xcell Electroporation System (Bio-rad, USA) under the conditions of 2,400 V, 25 μF, and 200 Ω. The transformed *A. tumefaciens* cells were spread on LB agar containing 100 μg/mL kanamycin and incubated at 30°C for 2 days. Colonies carrying pTRV2::transgene were selected by diagnostic PCR.

#### 2.2.2 Construction of *R. pseudosolanacearum* genomic library

The genomic library was constructed through the cloning of randomly sheared *R. pseudosolanacearum* genomic DNA fragments into the pTRV2 vector. The genomic DNA from *R. pseudosolanacearum* underwent shearing in an ultrasonicator twice, alternating between 30 secs of sonication and 30 secs of rest. Following purification, the sheared genomic DNA fragments were inserted into the SmaI-digested pTRV2 vector and subsequently transformed into *E. coli* DH5α cells. Colonies harboring the cloned pTRV2 vector were identified on LB agar plates containing 100 μg/mL kanamycin. Plasmids were then extracted and introduced into *A. tumefaciens* by electroporation as described above. *A. tumefaciens* possessing the cloned pTRV2 vector were selected on LB agar containing 100 μg/mL kanamycin and used for further experiments.

### 2.3 Silencing of virulence factors by PIBGS

The cotyledons of 2-week-old *N. benthamiana* were co-infiltrated with two different *A. tumefaciens* strains: one carrying pTRV1 and the other carrying pTRV2::transgene, each adjusted to an OD_600_ of 1. As a negative control, *A. tumefaciens* harboring pTRV2::GFP was employed. Two weeks after agroinfiltration, *N. benthamiana* plants were drenched with 30 mL of *R. pseudosolanacearum* (OD_600_ = 0.05), which had been cultivated on Casamino acid-Peptone-Glucose (CPG; 0.1% casamino acids, 1% peptone, and 0.5% glucose) agar plates at 30°C for 2 days. Symptoms of bacterial wilt in *N. benthamiana* were monitored from 10 to 14 days post-drenching, and disease severity was assessed using an in-house scoring index.

### 2.4 sRNA extraction

*N. benthamiana* leaves were infiltrated with *A. tumefaciens* carrying pTRV1 and pTRV2 harboring transgenes, as described above. Two weeks post-infiltration, the leaves were harvested, immediately immersed in liquid nitrogen, and ground to a fine powder using a mortar and pestle. The ground tissue was then stored at -80°C until further processing. The sRNAs were extracted as previously described (Rosas-Cardenas Fde et al., 2011). Briefly, the frozen leaf powder was transferred to a 1.5 mL microcentrifuge tube, to which 500 μL of LiCl extraction buffer (100 mM Tris-HCl, pH 9.0; 1% SDS; 100 mM LiCl; 10 mM EDTA) and 500 μL of phenol (pH 8.0) were added. The samples were thoroughly mixed by vortexing, heated at 60°C for 5 minutes, and then centrifuged at 13,000 rpm for 10 minutes at 4°C. The upper phase was carefully transferred to a new tube and heated for 15 minutes at 65°C. Subsequently, 50 μL of 5 M NaCl and 63 μL of 40% polyethylene glycol 8000 were added and mixed well by vortexing, and the mixture was incubated on ice for 1 hour. After centrifugation at 13,000 rpm for 10 minutes at 4°C, the supernatant, which contained the sRNAs, was transferred to a new tube. The sRNAs were precipitated by adding 50 μL of 3 M sodium acetate (pH 5.2) and 1,200 μL of absolute ethanol, followed by overnight incubation at -20°C. The next day, the pellet was air-dried, and resuspended in 20 μL of RNase-free water.

### 2.5 Stem-loop RT-PCR

To identify potential siRNA sequences in *N. benthamiana* infiltrated with *A. tumefaciens* harboring pTRV2::*xpsR-2*, we first predicted all possible 22-nt siRNA sequences and filtered them to include only those with a GC content between 30% and 50%. The most effective siRNAs were then selected and detected from both plant and bacterial cells using stem-loop RT-PCR, a sensitive method for siRNA quantification (**Supplementary Figure S2A**) To detect predicted siRNAs, sRNAs were extracted as previously described and four siRNA-specific cDNAs were reverse-transcribed using the PrimeScript RT Reagent Kit (Takara Bio) with 50 nM stem-loop RT primers (**Supplementary Table S2**). The synthesized cDNA was used as a template in RT-qPCR with siRNA-specific forward primers and a universal reverse primer (**Supplementary Table S2**). The RT-qPCR conditions were set as follows: initial denaturation at 95°C, followed by 40 cycles of 95°C for 10 seconds (denaturation), 60°C for 30 seconds (annealing), and 72°C for 30 seconds (elongation). The expression of the siRNAs was quantified by Cq values.

### 2.6 Off-target analysis

To predict potential off-targets in *R. pseudosolanacearum* strain SL1931 by plant-generated siRNAs, the sequence identity between the *R. pseudosolanacearum* strain SL1931 genome and the siRNA sequences was analyzed using a BLAST search. Off-target genes were specifically identified by locating at least 17-nt continuous complementarity within the 22-nt siRNA sequences, allowing for one mismatch or gap. Additionally, to identify potential off-targets in closely related species of *R. pseudosolanacearum*, a phylogenetic analysis of *xpsR* genes was conducted, including *R. pseudosolanacearum* and other related bacteria. Potential off-targets in these related species were predicted using the same criteria.

### 2.7 Screening of novel virulence genes by PIBGS

A total of 20,000 *A. tumefaciens* colonies, each harboring distinct pTRV2 vectors containing various genomic fragments from *R. pseudosolanacearum*, were screened to identify novel virulence factors. *A. tumefaciens* clones were grown in 96-well microplates filled with LB media for 2 days and twelve clones from the same row were pooled in a single tube for efficient screening. Subsequently, a mixture of pooled *A. tumefaciens* (OD_600_ = 1) and *A. tumefaciens* carrying pTRV1 (OD_600_ = 1) was co-infiltrated into the cotyledons of 2-week-old *N. benthamiana* plants as described above. After two weeks of agroinfiltration, the soil was drenched with *R. pseudosolanacearum* (OD_600_ = 0.05) and disease severity was measured. Then, individual clones from pools that showed a significant reduction in bacterial wilt disease were screened again. Through this process, the three most effective clones were selected for further analysis. To identify the transgene sequence inserted into pTRV2 vectors of effective clones, the plasmid was extracted from *A. tumefaciens*, and the inserted gene fragment was sequenced using a specific primer set (**Supplementary Table S2**).

### 2.8 Construction of a gene-deficient mutant

To verify the authenticity of the identified novel virulence factors, *R. pseudosolanacearum* mutants deficient in the target gene were generated using the double crossover homologous recombination method. The upstream and downstream flanking regions of the target gene were amplified by diagnostic PCR using primers listed in **Supplementary Table S2.** Subsequently, the amplified gene fragments were ligated into a suicide vector (pK18mobsacB), which was then transformed into *E. coli* DH5α competent cells. Clones carrying the recombinant vector were screened on LB agar containing 100 μL/mg kanamycin by colony PCR. Following plasmid extraction, 10 ng of the plasmid was introduced into *R. pseudosolanacearum* cells via electroporation under conditions of 1.8 kV for 5 ms. The electroporated cells were recovered in CPG media after incubation for 2 h at 30°C with shaking at 180 rpm and spread on CPG agar with 100 μl/mL kanamycin to facilitate the first crossover event for integration of the vector into the *R. pseudosolanacearum* genome. The colonies that had undergone the first crossover were incubated in CPG media without antibiotics with vigorous shaking at 30°C for 24 h to induce the second crossover event. The cells were then spread on CPG agar with 20% sucrose to select for cells that had lost the pK18mobsacB vector. The mutants were selected by diagnostic PCR using a specific primer set (**Supplementary Table S2**).

### 2.9 Evaluation of gene silencing efficacy

*R. pseudosolanacearum* or *Burkholderia vietnamiensis* was treated with 100 ng of sRNAs targeting virulence genes, extracted from *N. benthamiana* leaves. The bacteria and sRNAs were incubated together with agitation at 25°C for 8 hours. Following incubation, bacterial cells were lysed using acid-washed glass beads, and total RNA was extracted employing the RNeasy Plus Mini Kit (Qiagen) according to the manufacturer’s instructions. To remove any contaminating genomic DNA, the TURBO DNA-free™ Kit (Invitrogen™) was utilized. Subsequently, cDNA was synthesized from 1 μg of the extracted total RNA using the PrimeScript RT Reagent Kit (Takara Bio). The effectiveness of gene silencing by sRNAs was assessed using iQ™ SYBR® Green Supermix (Bio-Rad) on the CFX Opus 96 Real-Time PCR System (Bio-Rad, Hercules, CA, USA) using a specific primer set (**Supplementary Table S2**). The RT-qPCR conditions were as follows: an initial denaturation at 95°C, followed by 40 cycles of 95°C for 10 seconds (denaturation), 60°C for 30 seconds (annealing), and 72°C for 30 seconds (elongation). Gene expression levels were normalized to the housekeeping gene *gyrB*.

### 2.10 Observation of GFP signal in bacteria

sRNAs were extracted from *N. benthamiana* leaves infiltrated with *A. tumefaciens* carrying pTRV2::GFP as described above and then used to treat various genera of GFP-expressing bacteria, including *Acinetobacter baumannii*, *Bacillus subtilis*, *E. coli*, *Pectobacterium carotovora *subsp. *carotovora* (*Pcc*), *R. pseudosolanacearum*, *Staphylococcus aureus*, and *Xanthomonas axonopodis* pv. *vesicatoria* (*Xav*). Following a 3-day co-incubation period with the sRNAs, changes in bacterial GFP fluorescence were observed using the EVOS M5000 cell imaging system.

## 3 Results

### 3.1 Optimization of the PIBGS system

**Figure 1:**
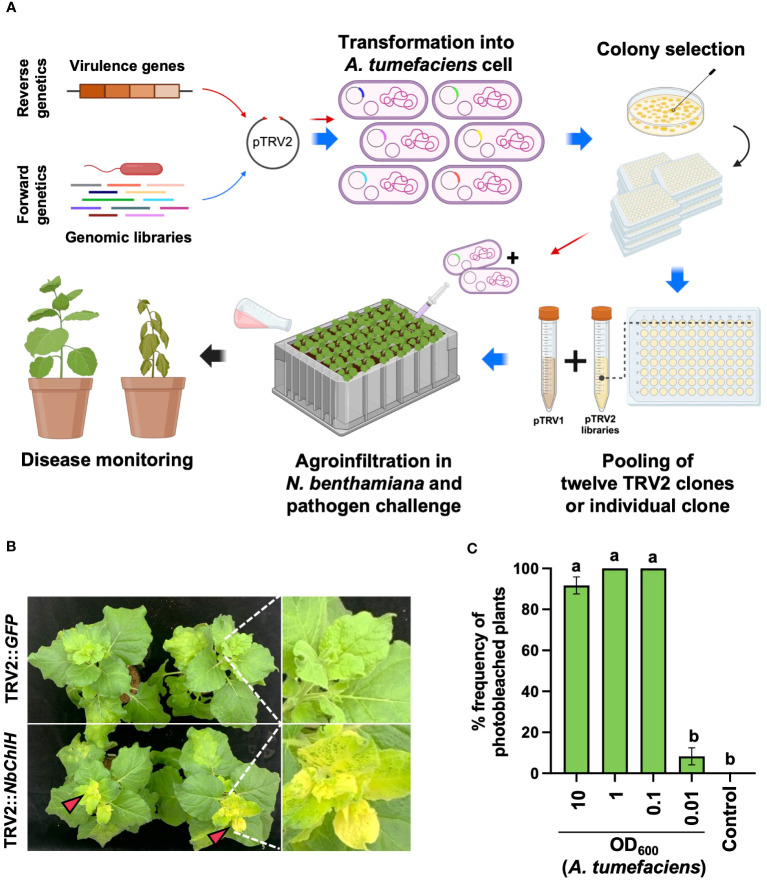
Plant-induced bacterial gene silencing. **(A)** Illustration of the experimental scheme for employing host-induced gene silencing (HIGS)-based plant-induced bacterial gene silencing (PIBGS) to silence bacterial virulence factors or identify those of *R. pseudosolanacearum*. **(B)** Verification of gene silencing efficiency through agroinfiltration of the *ChlH* gene into *N. benthamiana*, with successful silencing indicated by photobleached leaves in *NbChlH* gene-silenced plants. **(C)** Assessment of the frequency of photobleached plants following agroinfiltration, which depended on the OD_600_ of the *A. tumefaciens* culture. Different letters indicate statistically significant differences (*P* < 0.05). Statistical significance was determined using a Kruskal–Wallis test with Bonferroni correction. Error bars indicate SDs.

To evaluate the efficiency of the PIBGS system, a reverse genetics approach was employed. *A. tumefaciens* carrying the pTRV2 vector with inserts of DNA fragments from known virulence genes of *R. pseudosolanacearum* was co-agroinfiltrated with *A. tumefaciens* harboring the pTRV1 vector into the cotyledons of *N. benthamiana* 7 days post-transplantation (**Figure 1A**). In addition, to identify novel virulence genes of *R. pseudosolanacearum* using the PIBGS system, randomly sheared genome fragments of *R. pseudosolanacearum* were cloned into the pTRV2 vector. Subsequently, *A. tumefaciens* containing pTRV1 and pTRV2 with insertions of random genomic fragments were co-agroinfiltrated into *N. benthamiana* cotyledons (**Figure 1A**). For high-throughput screening, pools of twelve *A. tumefaciens*, each harboring different pTRV2 clones, were pooled together and agroinfiltrated. Two weeks after agroinfiltration, the soil was drenched with 30 mL of *R. pseudosolanacearum* (OD_600_ = 0.05), and the severity of bacterial wilt disease was monitored at 10 days post-challenge (**Figure 1A**).

The agroinfiltration conditions were optimized by silencing the *chlH* gene in *N. benthamiana*, which encodes the magnesium-chelatase subunit. Silencing results in a phenotypic change from green leaves to yellowish leaves (**Figure 1B**). Plants infiltrated with *A. tumefaciens* harboring pTRV2::GFP (control) exhibited no color change in their leaves, whereas all leaves were photobleached following infiltration with *A. tumefaciens* carrying pTRV2::*NbChlH* at an OD_600_ of 0.1 (**Figures 1B, C**). Therefore, an OD_600_ of 1 was chosen as the agroinfiltration condition for the induction of PBIGS in this study.

### 3.2 Efficiency of PIBGS in the soil-borne phytopathogen *R. pseudosolanacearum*

**Figure 2:**
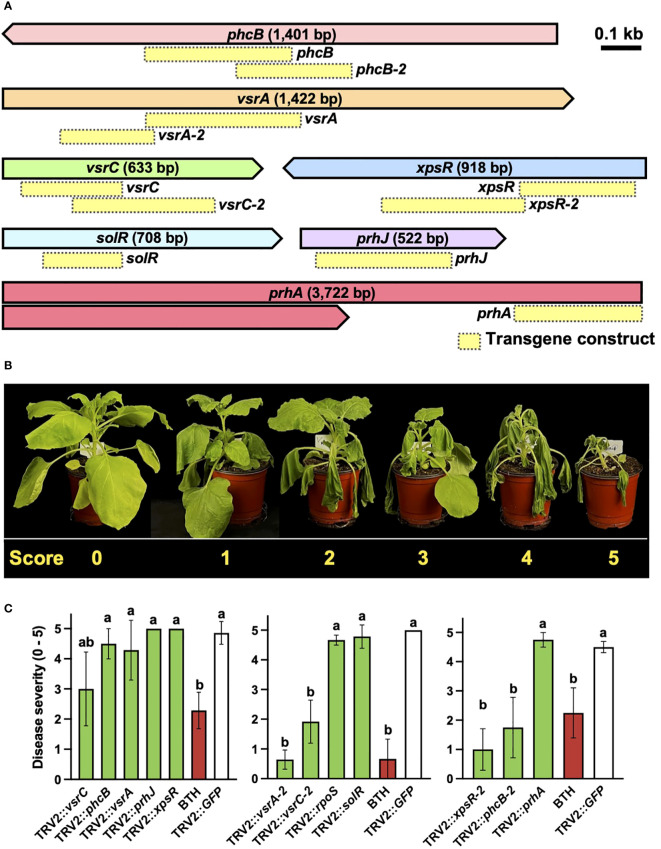
Silencing of *R. pseudosolanacearum* virulence factors through plant-induced bacterial gene silencing. **(A)** Presentation of the target genes and the configurations of the transgene constructs utilized in this study. Yellow boxes indicate transgene constructs. **(B)** Disease severity rating of bacterial wilt: 0, no symptoms; 1, 1–20% of leaves wilted; 2, 21–40% of leaves wilted; 3, 41–60% of leaves and petioles wilted; 4, 61–80% of leaves and petioles wilted; and 5, 81–100% of leaves, petioles, and stems wilted. **(C)** Severity of bacterial wilt in plants agroinfiltrated with constructs targeting known virulence factors. Different letters indicate statistically significant differences (*P* < 0.05). Statistical significance was determined using a Kruskal–Wallis test with Bonferroni correction. Error bars indicate SDs.

A reverse genetics approach was employed to demonstrate the effectiveness of PIBGS with known virulence factors (**Figure 1A**). Each of the eight gene fragments targeting previously identified virulence factors *phcB, prhA, prhJ, rpoS, solR, vsrA, vsrC*, and *xpsR* was cloned into pTRV2 (**Figure 2A**; **Supplementary Tables S1**). At 14 days post-agroinfiltration, the roots were drench-inoculated with 30 mL of an *R. pseudosolanacearum* (OD_600_ = 0.05) suspension and disease severities were scored (**Figure 2B**). Plants treated with *A. tumefaciens* carrying pTRV2::GFP displayed severe wilt (**Figure 2C**). By contrast, those infiltrated with *A. tumefaciens* harboring pTRV2:: *phcB-2*, pTRV2::*vsrA-2*, pTRV2::*vsrC-2*, or pTRV2::*xpsR-2* showed reduced bacterial wilt severity by 78%, 61%, 87%, and 62%, respectively, compared with the control (**Figure 2C**). For the genes *phcB* (1,401 bp), *vsrA* (1,442 bp), and *vsrC* (663 bp), the efficiency of gene silencing differed based on the regions targeted by transgene constructs (**Figures 2A, C**), suggesting that the region of a gene targeted by siRNA can influence its silencing effect in bacteria. These results conclusively show that PIBGS is effective in bacteria, although the efficiency depends on the siRNA employed and the RNA silencing mechanism remains unclear.

### 3.3 Screening of novel virulence factors by PIBGS

**Figure 3:**
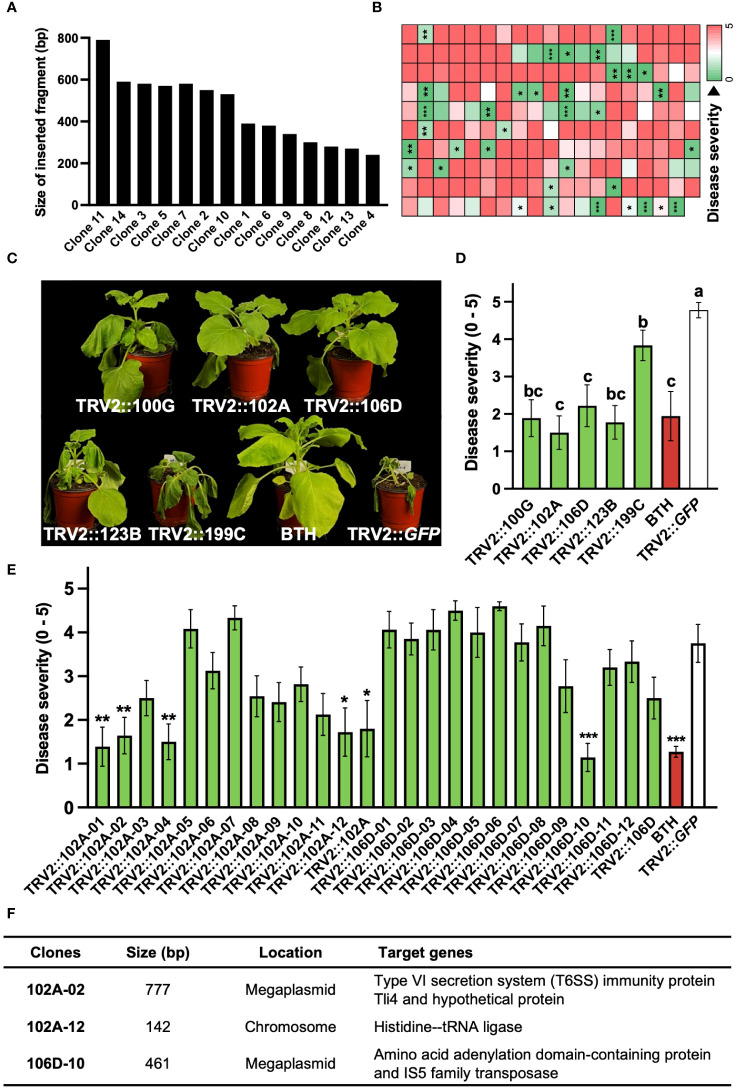
Identification of novel virulence factors of *R. pseudosolanacearum* via plant-induced bacterial gene silencing. **(A)** Characterization of DNA fragment sizes cloned into the pTRV2 vector by sequencing. **(B)** Results of first-round screening of pTRV2 clone pools carrying randomly sheared *R. pseudosolanacearum* DNA fragments. Each box indicates the severity of bacterial wilt in plants infiltrated with different clone pools. **(C)** Photographs taken 14 days after *R. pseudosolanacearum* infection. **(D, E)** Second-round screening of pTRV2 clone pools **(D)** and third-round screening of individual clones from the 102A and 106D pools **(E)**, based on the severity of bacterial wilt in agroinfiltrated plants. **(F)** Summary of the identified clones. Statistical significance was analyzed by the Mann–Whitney U test **(B, E)** or Kruskal–Wallis test with Bonferroni correction **(D)**. Error bars indicate SDs. Asterisks denote a significant difference compared to the TRV2::GFP group (*, P < 0.05; **, P < 0.005; ***, P < 0.0005).

PIBGS was also utilized to identify new virulence factors in *R. pseudosolanacearum.* In contrast to targeted mutagenesis in bacteria, PIBGS can precisely silence specific genes using bacterial DNA fragments cloned into pTRV2, albeit in a random manner (**Figure 1A**). This approach offers a cost-effective and efficient strategy for discovering novel virulence factors through a positive selection approach and offers advantages over traditional random mutagenesis, which requires individual validation of bacterial virulence factors. Following the cloning of *R. pseudosolanacearum* genomic fragments into the pTRV2 vector, each clone harbored random genomic fragments of varying sizes (**Figure 3A**). In this study, twelve *A. tumefaciens* clones carrying pTRV2 with different bacterial genomic fragments were pooled and agroinfiltrated to identify novel virulence factors in *R. pseudosolanacearum* (**Figure 1A**). First-round high-throughput screening yielded 35 pools that significantly reduced bacterial wilt severity upon *R. pseudosolanacearum* infection (**Figure 3B**). Subsequently, a second-round of screening was conducted with the top five pools (**Figures 3C, D**). Among the five pools tested, pTRV2::102A and pTRV2::106D, which reduced the severity of bacterial wilt by 57% and 58%, respectively, compared with the pTRV2::GFP control, were selected for further screening of individual clones. As a result, plants infiltrated with six single clones exhibited reduced bacterial wilt symptoms similar to those of positive control plants pretreated with 1 mM benzothiadiazole (BTH), a chemical plant protectant that induces systemic acquired resistance (**Figure 3E**). A third-round of screening with each single clone resulted in the selection of three novel virulence factors (**Figure 3F**). Sequencing analysis of the inserted DNA fragments revealed that the sequences of the transgenes from 102A-02 target genes overlapped with those encoding the type VI secretion system (T6SS) immunity protein Tli4 and a hypothetical protein encoded by a megaplasmid in *R. pseudosolanacearum*. Furthermore, 102A-12 specifically targeted the gene encoding histidine—tRNA ligase in the chromosome, and 102D-10 targeted genes encoding an amino acid adenylation domain-containing protein and an IS5 family transposase in the megaplasmid. While T6SS is a recognized virulence factor of *R. solanacearum* (Zhang et al., 2014), other factors targeted by 102A-12 and 102D-10 have not been reported previously.

### 3.4 Validation of virulence factors identified by PIBGS

**Figure 4:**
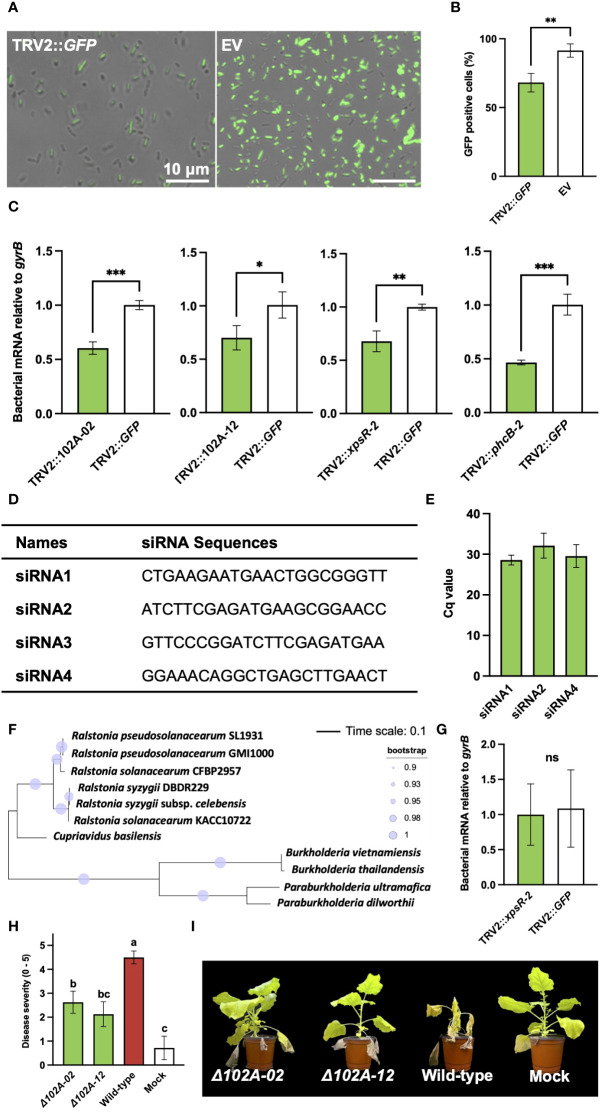
Validation of virulence factors identified through plant-induced bacterial gene silencing and the efficiency of gene silencing in bacterial cells. **(A)** Observation of fluorescence signals in *R. pseudosolanacearum* expressing GFP after treatment with siRNA derived from leaves infiltrated by *A. tumefaciens* carrying either the pTRV2::GFP vector or an empty pTRV2 vector (EV). **(B)** Proportion of GFP-positive *R. pseudosolanacearum* cells co-incubated with siRNAs from leaves infiltrated by *A. tumefaciens* carrying either pTRV2::GFP or the pTRV2 vector. **(C)** Expression levels of target genes in *R. pseudosolanacearum* following *in vitro* treatment with siRNA derived from leaves infiltrated with *A. tumefaciens* harboring transgene constructs. **(D)** Four siRNA sequences were selected based on a GC content range of 30 to 50%. **(E)** Result of stem-loop RT-PCR showing detection of three siRNAs from tobacco leaves infiltrated with *Agrobacterium* harboring pTRV2::*xpsR-2*. **(F)** Phylogenetic tree of *xpsR*-homologous genes *in R. pseudosolanacearum* and related bacterial species. **(G)** Expression level of the *xpsR*-homologous gene in *B. vietnamiensis* following treatment with sRNAs extracted from plant leaves **(H)** Comparison of bacterial wilt severity in plants inoculated with either wild-type or mutant strains of *R. pseudosolanacearum*. **(I)** Plant photographs captured 20 days post-infection with wild-type or gene-deficient mutants of *R. pseudosolanacearum*. Asterisks denote a significant difference between the two groups (ns, not significant; *: P < 0.05; **: P < 0.005; ***: P < 0.0005).

To prove that the diminished virulence of *R. pseudosolanacearum* was directly attributable to PIBGS, siRNAs produced in plant leaves targeting GFP were applied to GFP-expressing *R. pseudosolanacearum*, and the gene silencing efficacy within bacterial cells was assessed. The results revealed that fluorescence signal intensity of *R. pseudosolanacearum* cells after co-incubation *in vitro* for 3 days with siRNAs from *N. benthamiana* leaves infiltrated by *A. tumefaciens* with the pTRV2::GFP vector was 27.8% lower than in cells treated with siRNAs from leaves infiltrated with an empty vector (*P* = 0.009) (**Figures 4A, B**). Subsequently, *R. pseudosolanacearum* was incubated with siRNAs extracted from *N. benthamiana* leaves post-infiltration with *A. tumefaciens* carrying pTRV2::102A-02, pTRV2::102A-12, pTRV2::*xpsR-2*, or pTRV2::*phcB-2* for 8 hours to explore the silencing of target genes. Unlike *R. pseudosolanacearum* treated with siRNAs extracted from *N. benthamiana* leaves infiltrated with pTRV2::GFP, siRNAs from leaves infiltrated with pTRV2 carrying virulence gene fragments significantly silenced their target genes in *R. pseudosolanacearum* (*P* = 0.0006, 0.034, 0.0053, 0.0007 for pTRV2::102A-02, pTRV2::102A-12, pTRV2::*xpsR-2*, or pTRV2::*phcB-2*, respectively) (**Figure 4C**).

To determine specific siRNAs for silencing bacterial genes, potential siRNAs generated from tobacco leaves infiltrated with *A. tumefaciens* harboring the pTRV2::*xpsR-2* construct were analyzed, given their strong gene silencing effect when treated in *R. pseudosolanacearum* (**Figure 2C**). All possible 22-nt sequences producible from pTRV2::*xpsR-2* were predicted and filtered based on a GC content range of 30-50% (**Supplementary Figure S2A**). Four siRNAs with 50% of GC content were selected for further experiments (**Figure 4D**). The presence of these four siRNAs was detected using stem-loop RT-PCR, and three out of the four siRNAs were detected in tobacco leaves (**Figure 4E**; **Supplementary Figure S2A**). However, these siRNAs were not detected in RNAs extracted from root-colonizing bacterial cells, potentially due to their low abundance, making detection challenging. In addition, a BLAST search was used to predict potential off-target effects of these three siRNAs on *R. pseudosolanacearum* strain SL1931 (**Supplementary Figure S2A**). Off-target genes were identified based on a minimum of 17-nt continuous complementarity with the 22-nt siRNA sequences, allowing for one mismatch or gap. As a result, none of the three siRNAs showed predicted off-targets (**Supplementary Figure S2B**). Potential off-target effects on the *xpsR*-homologous genes of closely related bacteria, *Cupriavidus basilensis* and *Burkholderia vietnamiensis*, were also analyzed (**Figure 4F**). No potential off-targets were found in those bacterial species as well (**Supplementary Figure S3B**). Indeed, siRNAs extracted from *N. benthamiana* leaves infiltrated with pTRV2::*xpsR*-*2* did not reduce the expression level of *xpsR*-homologous gene in *B. vietnamiensis* (**Figure 4G**). These results demonstrate that PIBGS can specifically and accurately silence target bacterial genes through cross-kingdom RNAi facilitated by siRNAs generated in plants.

To verify the authenticity of the virulence factors identified by PIBGS, gene-deficient mutants of 102A-02 and 102A-12 were constructed. Both knock-out mutants displayed lower virulence than wild-type *R. pseudosolanacearum*, with reductions in bacterial wilt of 41.7% and 52.8%, respectively, confirming the efficacy of PIBGS in uncovering novel virulence genes in soil-borne phytopathogenic bacteria (**Figures 4H, I**).

### 3.5 Potential applications of PIBGS in diverse bacterial genera

**Figure 5:**
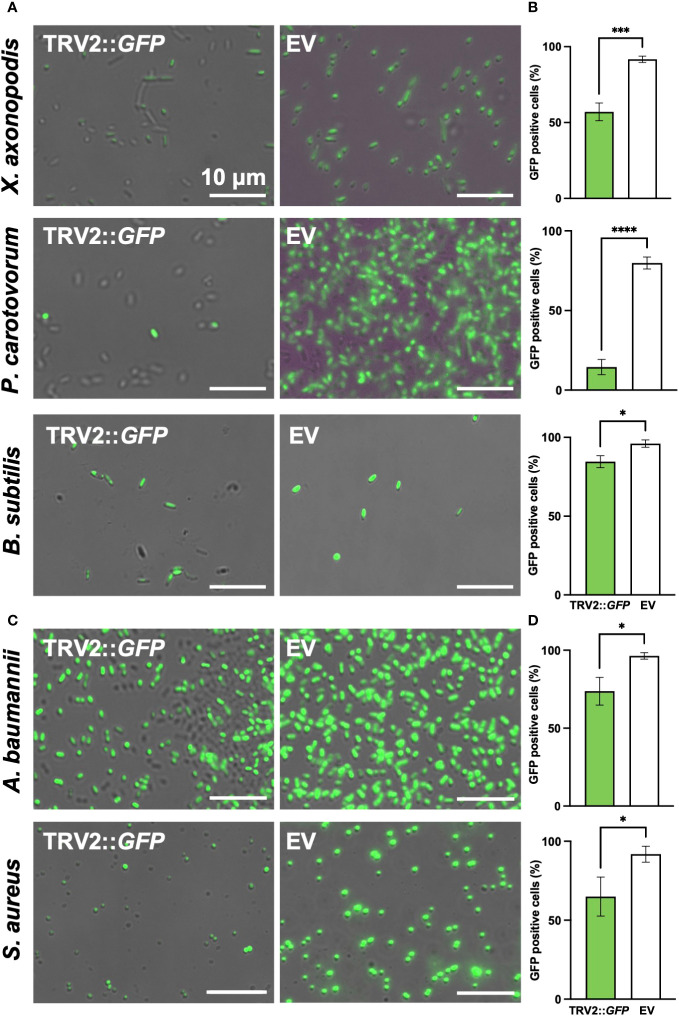
GFP signal observation in various bacterial genera post-siRNA treatment **(A)** Fluorescence signals were observed in the phytopathogens *Xanthomonas axonopodis* pv. *vesicatoria* (*Xav*) and *Pectobacterium carotovorum* subsp. *carotovorum* (*Pcc*), as well as in the **(C)** clinical pathogens *Acinetobacter baumannii* and *Staphylococcus aureus* and **(B)** the plant growth-promoting rhizobacteria *Bacillus subtilis*, following treatment with siRNA derived from leaves infiltrated by *A. tumefaciens* carrying either the pTRV2::GFP vector or an empty pTRV2 vector (EV). **(B, D)** Relative abundance of GFP-positive bacterial cells. Asterisks denote a significant difference between the two groups (*: P < 0.05; ***: P < 0.0005; ****: P < 0.0001).

To demonstrate the versatility of PIBGS across various phytopathogens, siRNAs targeting GFP, derived from *N. benthamiana* leaves, were treated to *Xanthomonas axonopodis* pv. *vesicatoria* (*Xav*) and *Pectobacterium carotovorum* subsp. *carotovorum* (*Pcc*), the causative agents of bacterial spot disease and bacterial soft rot disease, respectively. Following siRNA application, the GFP signal in *Xav* displayed a slight reduction, with the majority of bacteria retaining fluorescence, although a notable number lost their fluorescence (*P* = 0.0007) (**Figures 5A, B**). By contrast, *Pcc* showed a substantial decrease in fluorescence post-siRNA treatment (*P* < 0.0001) (**Figures 5A, B**). Furthermore, siRNAs targeting GFP were also applied to clinically relevant pathogens, including the Gram-negative bacteria *Acinetobacter baumannii* and *Escherichia coli*, as well as the Gram-positive bacteria *Staphylococcus aureus*. The treatment led to decreases in the GFP signal in *A. baumanii* and *S. aureus* (*P* = 0.0124 and 0.0249, respectively), whereas the gene silencing effect in *E. coli* was not significant (**Figures 5C, D**; **Supplementary Figure S3**). The silencing effects of siRNAs on GFP expression were also validated in the plant growth-promoting rhizobacterium, *B. subtilis* (*P* = 0.0111) (**Figures 5A, B**). These results suggest that PIBGS has the potential to be broadly effective against both Gram-negative and Gram-positive bacteria, including phytopathogens and clinical pathogens, although its efficacy may differ between bacterial species.

## 4 Discussion

Although previous studies have shown that cross-kingdom small RNA communication between eukaryotes and prokaryotes is ubiquitous (Knip et al., 2014), the use of cross-kingdom RNAi for controlling infection by prokaryotic phytopathogens remains unexplored. Our study marks the first successful attempt at reducing the virulence of the soil-borne phytopathogen *R. pseudosolanacearum* through the use of HIGS-based bacterial gene silencing, referred to as PIBGS. Our results revealed a significant reduction of bacterial wilt in *N. benthamiana* following infiltration with *Agrobacterium* carrying TRV harboring bacterial transgenes by the silencing of virulence genes in *R. pseudosolanacearum*. The release of siRNAs produced in plant cells by TRV into the external environment and their subsequent uptake by bacterial cells leads to effective targeted gene silencing. These findings are consistent with numerous studies showing that siRNAs transiently produced in plants through VIGS or HIGS can effectively silence genes in various eukaryotic pathogens, such as fungi, oomycetes, and insects. However, a limitation of the current study is that the molecular mechanisms governing the transfer of plant-derived siRNAs into bacterial cells were not explored. The mechanism responsible for the subsequent silencing of the target mRNA also remains unclear.

While this study does not provide experimental evidence for the transfer mechanism of siRNA from plant to bacteria via PIBGS, there are several proposed mechanisms for the transfer of plant-derived siRNAs to eukaryotic pathogens. These mechanisms include haustoria-mediated transfer, direct uptake, and phloem-mediated transport, all of which contribute to the complex process of siRNA movement from plants to pathogens (Chen and Rechavi, 2022) (**Supplementary Figure S4**). However, delivering siRNA into bacterial cells via PIBGS poses a challenge since bacteria lack structures like haustoria and mouths, which are present in eukaryotic pathogens. Additionally, although sRNAs are primarily synthesized within plant cells, recent studies detect them in apoplastic fluid suggest a potential mechanism for their movement between plant cells (Zand Karimi et al., 2022). The apoplastic fluid of plants contains numerous EVs (Baldrich et al., 2019; He et al., 2021). The observed presence of EVs in apoplastic fluid suggests that they may be involved in the encapsulation of sRNAs, potentially facilitating cross-kingdom gene silencing through their transfer to diverse organisms. Indeed, EVs are known for their ability to encapsulate and transport functional molecules to recipient cells across various kingdoms. To ensure sRNAs are transferred to other organisms without degradation, they must be either closely associated with RNA-binding proteins or encapsulated within EVs. This arrangement provides protection against extracellular enzymes and maintains their integrity (Cai et al., 2018b, Cai et al., 2018c; He et al., 2021). The transport of sRNAs via EVs has been reported in several plant species; for instance, plants such as *Arabidopsis*, cotton, and wheat utilize EVs to transfer defensive siRNAs to the fungal pathogens *Botrytis cinerea*, *Verticillium dahlia*, and *F. graminearum* as well as the oomycete pathogen *Phytophthora capsici*, resulting in the effective silencing of the targeted virulence genes (Prosser et al., 2011; Jiao and Peng, 2018; Cai et al., 2018b; Hou et al., 2019). Similarly, a recent study in *N. benthamiana* successfully isolated EVs from the apoplastic fluid, which revealed the accumulation of sRNAs within these vesicles (He et al., 2021). Furthermore, the isolation of EVs from root exudates of plants grown hydroponically indicates that EVs are generally released from plant roots as well (De Palma et al., 2020). These observations suggest a plausible mechanism for our findings that EVs encapsulate siRNAs targeting bacterial genes, are released from *N. benthamiana* roots, and deliver siRNAs to the root-associated phytopathogen *R. pseudosolanacearum*.

In eukaryotic plant pathogens, the siRNAs transported from plant can effectively silence specific genes by hijacking the host’s RNAi machinery (Cai et al., 2018a). This process begins when the transferred siRNAs are incorporated into the RISC, a multicomponent protein assembly. A critical component of RISC is the Argonaute protein (AGO), which not only binds siRNAs but also has the capacity to cleave RNA. Once integrated into the RISC, the siRNA serves as a guide, leading the complex to target mRNAs either to inhibit their translation or directly cleave them. Cleavage of mRNA results in fragments that can act as templates for dsRNA production, which is facilitated by proteins belonging to the RNA-dependent RNA Polymerase (RdRP) family. This initiates a cascading cycle of RNAi, generating secondary siRNAs and amplifying the silencing effect (Shabalina and Koonin, 2008).

**Figure 6:**
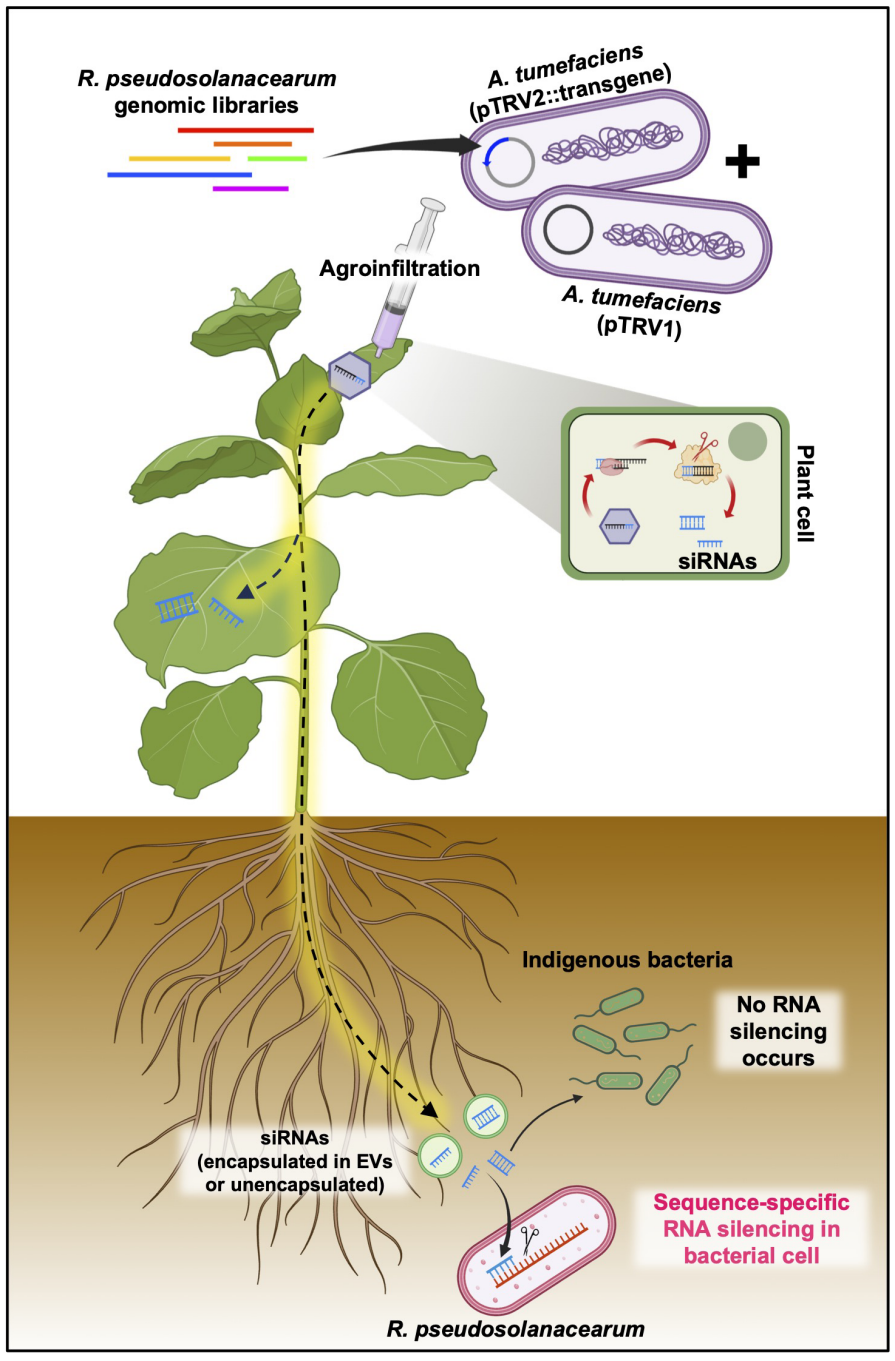
Putative mode of action of plant-induced bacterial gene silencing. Transgene constructs designed to target *R. pseudosolanacearum* virulence factors, cloned into the pTRV2 vector, and introduced into *N. benthamiana* via agroinfiltration are transformed into siRNAs through the host RNAi machinery. These siRNAs, which are capable of systemic distribution, have the potential to penetrate root-associated *R. pseudosolanacearum* cells, either encapsulated or unencapsulated within extracellular vesicles (EVs). Upon entry into the bacterial cells, the siRNAs facilitate the silencing of specific virulence factors by targeting mRNAs via as yet unidentified mechanisms.

However, the scenario is markedly different in bacteria. Currently, there is no evidence to support that bacteria possess the molecular mechanisms of RNAi triggered by dsRNAs and/or siRNAs derived from animals or plants due to their lack of the intricate RNAi machinery found in eukaryotes, including key components, such as RISC, AGO, and RdRP. While there have been reports of siRNA delivery into bacterial cells from mammals, experimental evidence that plant-derived EVs are delivered into bacterial cells remains scarce. Consequently, the detailed mechanisms by which plant root EVs are perceived by bacteria, and how plant siRNAs could potentially be adapted into a bacterial RNAi-like process, require further elucidation through advanced molecular and cell biology research (**Figure 6**).

In the mammalian gut, miRNAs are selectively delivered to gut-colonizing bacteria via exosome-like nanoparticles (ELNs) derived from ingested edible plants (Teng et al., 2018). Notably, ginseng ELNs are preferentially absorbed by Bacteroidales, while ginger ELNs favor Lactobacillaceae, and ELNs from garlic and grapefruit are taken up more by Ruminococcaceae bacteria. This selective uptake mechanism underpins our observations that *N. benthamiana*-derived siRNAs, specifically those targeting GFP, have varying efficiencies in diminishing GFP fluorescence across different bacterial taxa (**Figure 5**; **Supplementary Figure S3**). Although understanding the precise molecular mechanism of selective siRNA transfer by various plant species to distinct bacterial taxa remains will require further study, the effectiveness of sRNA transfer has been convincingly demonstrated using nano vectors constructed from ginseng ELN-derived lipids. Importantly, the presence of phosphatidic acid in ELN lipids is pivotal in enhancing their efficient uptake by bacteria and in regulating the duration and quantity of ELN accumulation within the gut. Given that phosphatidic acid is a major lipid component of plant EVs, as evidenced by its presence in the EVs of sunflower apoplastic fluid, grapes, oranges, and ginger (Alfieri et al., 2021; Urzì et al., 2021), it is plausible that EVs carrying siRNAs secreted from *N. benthamiana* could also be transferred to root-associated bacteria.

Furthermore, recent studies have indicated that plant EVs offer innovative therapeutic approaches for tissue regeneration, counteracting inflammation, and tumor progression via their ability to regulate the complicated gene expression involved in inflammation, tumor progression, and tissue repair (Yang et al., 2023). Our results showing that siRNAs extracted from *N. benthamiana* can silence genes across diverse bacterial taxa underscore the potential of plant EVs and plant-derived siRNAs for controlling both Gram-negative and Gram-positive pathogenic bacteria by silencing species-specific virulence genes. This strategy is expected to be capable of inhibiting selectively specific bacterial species within complex bacterial communities, such as the gut microbiome.

## 5 Conclusion

In conclusion, our study is the first to demonstrate plant-induced gene silencing in bacteria, offering an efficient strategy for controlling the soil-borne phytopathogen *R. pseudosolanacearum*. This new method could significantly contribute to identifying a multitude of phytobacterial virulence factors and managing bacterial infections. Moreover, this tool can be applied in field conditions, enabling precise gene-based control of bacterial pathogens without impacting indigenous microbial communities. Indeed, our results showed that siRNAs generated in plant did not exhibit off-target effects in *R. pseudosolanacearum* or other bacteria, though potential off-target pitfalls are carefully considered. In addition, its application to medical therapeutics holds promise for the development of innovative treatment approaches.

## Data availability statement

The original contributions presented in the study are included in the article/**Supplementary Material**. Further inquiries can be directed to the corresponding author.

## Author contributions

SJ: Data curation, Investigation, Methodology, Validation, Visualization, Writing – original draft, Writing – review & editing. DK: Formal analysis, Investigation, Methodology, Visualization, Writing – original draft, Writing – review & editing. SL: Investigation, Methodology, Writing – original draft. C-MR: Conceptualization, Funding acquisition, Project administration, Supervision, Visualization, Writing – original draft, Writing – review & editing.
