# Supplementary material for: Plant-induced bacterial gene silencing: a novel control method for bacterial wilt disease
Source: Front Plant Sci. 2024 Aug 2;15:1411837. doi: 10.3389/fpls.2024.1411837 (PMC11327017; doi:10.3389/fpls.2024.1411837)
Supplement: Supplementary file 1 [file DataSheet_1.pdf]

Figure S1

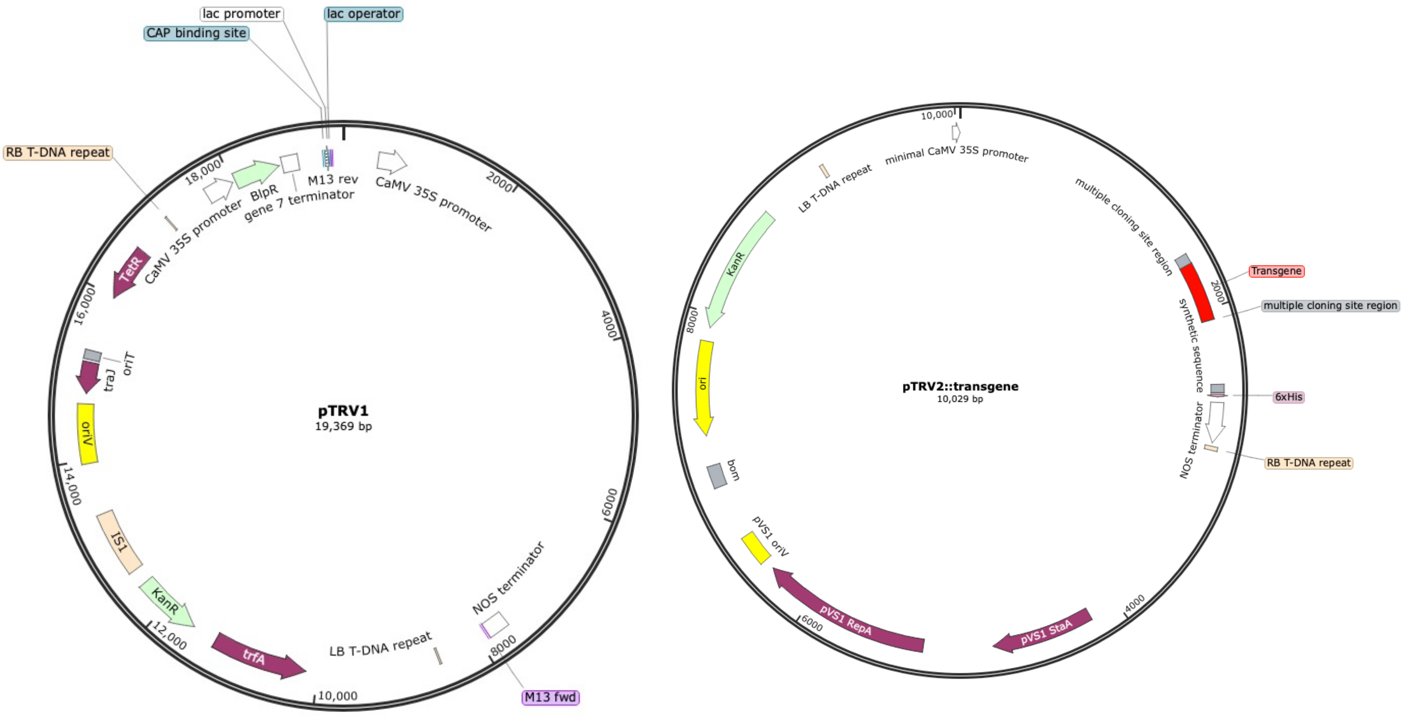

Figure S1. Plasmid maps of pTRV1 and pTRV2 containing the transgene construct.
Plasmid maps of pTRV1 and pTRV2 utilized in this study. The transgene construct is cloned into pTRV2. The red-colored segment in pTRV2 indicates transgene constructed inserted into the plasmid.

Figure S2

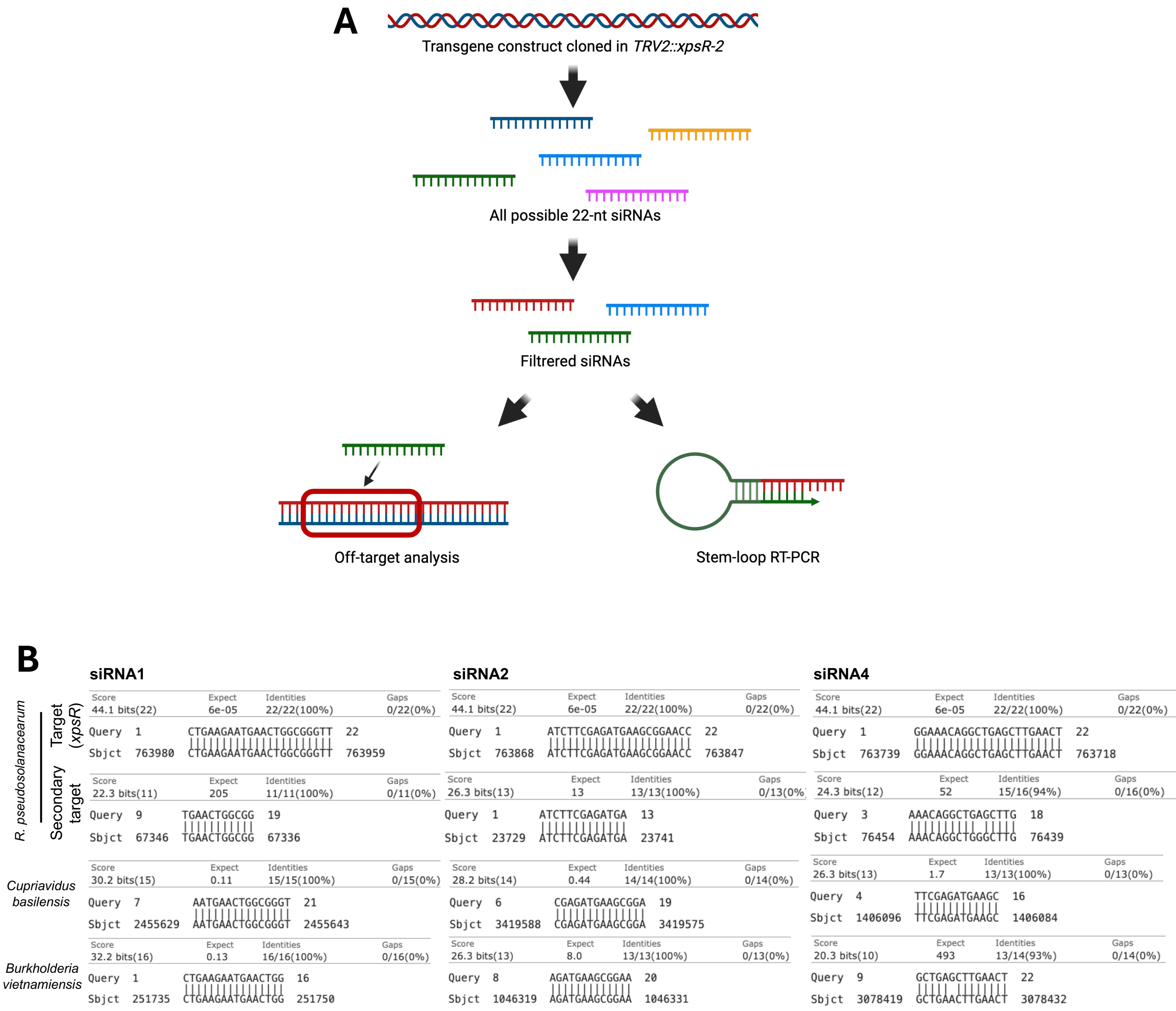

Figure S2. Detection of siRNAs by stem-loop RT-PCR
(**A**) Overview of the experimental scheme for siRNA sequence prediction, off-target analysis, and stem-loop RT-PCR. (**B**) Analysis of potential off-targets by siRNAs in *R. pseudosolanacearum*, *Cupriavidus basilensis*, and *Burkholderia vietnamiensis*. None of the siRNAs showed potential off-target effects

Figure S3

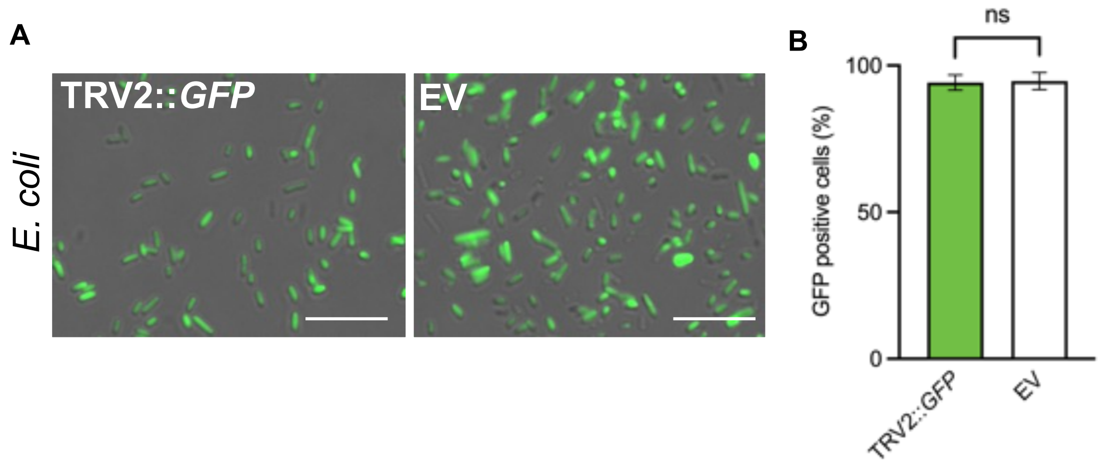

Figure S3. GFP signal observation in *Escherichia coli* post-siRNA treatment
(**A**) Fluorescence signals were observed in the clinical pathogens *E.coli* following treatment with siRNA derived from leaves infiltrated by *A. tumefaciens* carrying either the pTRV2::GFP vector or an empty pTRV2 vector (EV). (**B**) Relative abundance of GFP-positive bacterial cells.

Figure S4

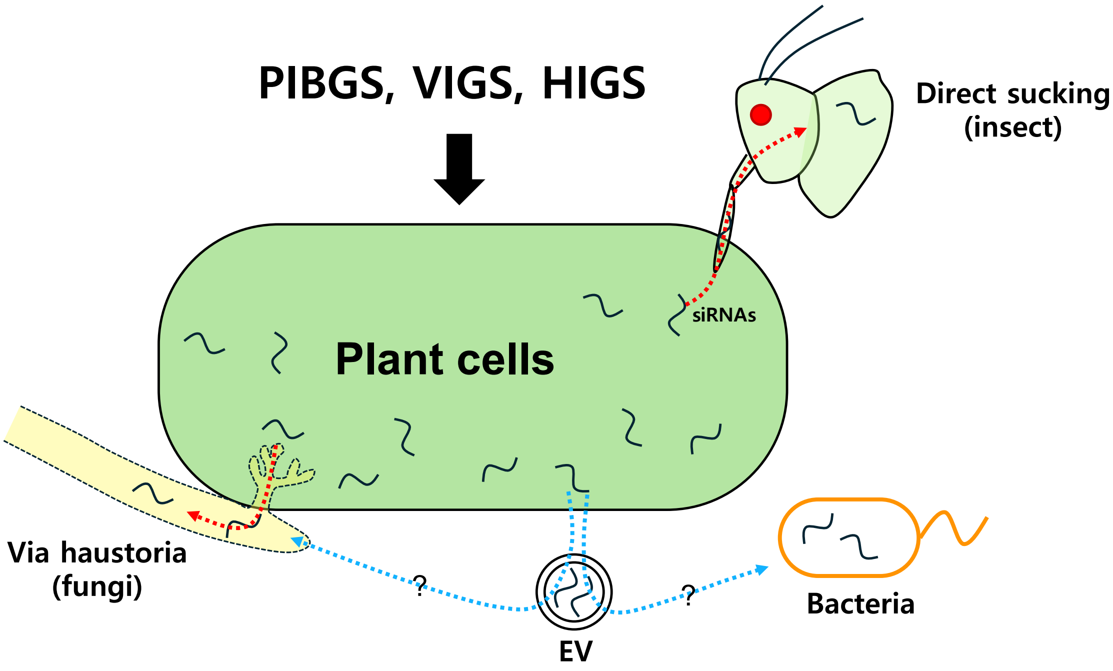

Figure S4. Description of siRNA delivery mechanism

siRNAs produced in plant cells via PIBGS, VIGS, or HIGS can be taken up by insects through feeding on the phloem and by fungi through specialized structures called haustoria. In bacteria, siRNAs can be taken up through extracellular vesicles (EVs). These EV-encapsulated siRNAs can also be delivered to fungi and other organisms.
