# Supplementary material for: Plant-induced bacterial gene silencing: a novel control method for bacterial wilt disease
Source: Front Plant Sci. 2024 Aug 2;15:1411837. doi: 10.3389/fpls.2024.1411837 (PMC11327017; doi:10.3389/fpls.2024.1411837)
Supplement: Supplementary file 2 [file DataSheet_2.pdf]

**Table S1. Information on virulence genes of *R. pseudosolanacearum* targeted in this study.**

| | Gene | Function |
|---|---|---|
| Quorum sensing | *phcB* | Biosyntheic enzyme for 3-OH PAME |
| | *solR* | LuxR-type transcriptional activator |
| Extracellular polysaccharide synthesis | *xpsR* | Novel transcriptional activator |
| | *vsrC* | Sensor response regulator |
| | *vsrA* | Sensor kinase |
| Controlling of *hrp* regulon | *prhA* | Surface receptor |
| | *prhJ* | LuxR/UhpA-type transcriptional activator |
| Others | *rpoS* | Alternate sigma factor |

**Table S2. Primers used in this study to amplify virulence genes**

| Name | Sequence | Purpose |
|---|---|---|
| phcB_F | tcacgcgtctcgaggcccACTACCTCGACTGGCTCGAA | Silencing of known virulence factors of *R. pseudosolanacearum* by PIBGS |
| phcB_R | tgtcttcgggacatgcccGCCGAGATAGTTGACCAGGA | |
| prhA_F | tcacgcgtctcgaggcccGTCGGCACATAGGGATTCTG | |
| prhA_R | tgtcttcgggacatgcccGCCGAGCGGTTCTACTACAC | |
| xpsR_F | tcacgcgtctcgaggcccGGACTGATGATCCAGGTGGT | |
| xpsR_R | tgtcttcgggacatgcccTTACTTTGCGGACCTGCTCT | |
| phcB2_F | tcacgcgtctcgaggcccCCCTTCGTCTATCGCACCTA | |
| phcB2_R | tgtcttcgggacatgcccCTGTACGCCATCCATCTCCT | |
| prhJ_F | tcacgcgtctcgaggcccGCCGAAAACCAGTATCTCCA | |
| prhJ_R | tgtcttcgggacatgcccCGATCTCCACATTGGATTTG | |
| vsrC2_F | tcacgcgtctcgaggcccACCACCCTCTCGCCTTATCT | |
| vsrC2_R | tgtcttcgggacatgcccGTGCTTTCGCGACGATTT | |
| xpsR2_F | tcacgcgtctcgaggcccAGAGCAGGTCCGCAAAGTAA | |
| xpsR2_R | tgtcttcgggacatgcccCGCGAACAATTGGACAAGTA | |
| vsrA_F | tcacgcgtctcgaggcccGAAACCGCCATCAATGAACT | |
| vsrA_R | tgtcttcgggacatgcccAGATCAGCGTGAGCTCCATC | |
| vsrA2_F | tcacgcgtctcgaggcccGAGATGGAGCTCACGCTGAT | |
| vsrA2_R | tgtcttcgggacatgcccGTCTTCTCGTCCTCGGTCAC | |
| vsrC_F | tcacgcgtctcgaggcccCTGCTGGATGTCTGGCTGT | |

| | | |
|---|---|---|
| vsrC_R | tgtcttcgggacatgcccATTTGTTCGGCAACCCTTC | |
| rpoS_F | tcacgcgtctcgaggcccACGTGGCATAGGTGGAGAAG | |
| rpoS_R | tgtcttcgggacatgcccATCGTCGATACTCCGGACAC | |
| solR_F | tcacgcgtctcgaggcccGTGTCCATCCGTGATGAGG | |
| solR_R | tgtcttcgggacatgcccCGTTGTTGACGTGGAAATTG | |
| vsrA-qF | AGATTGAGCAGGATGGTCGG | Validation of gene silencing in *R. pseudosolanacearum* |
| vsrA-qR | ACCAAGATGGACCTGCACTG | |
| xpsR-qF | GGTTGCAGGGTACGTTGCTC | |
| xpsR-qR | TGCTGCTCGAAGCGATGG | |
| vsrC-qF | GATTTGTTCGGCAGCCCTTC | |
| vsrC-qR | GTTCCCGTCGGAAGACGAC | |
| phcB-qF | TGCTGTACGCCATCCATCTC | |
| phcB-qR | GTTCAAGGTGCTGAACGTCG | |
| A02-qF | ATGCCCTGATCACGATCGAC | |
| A02-qR | TACTCCTCTGCCTCGATGGG | |
| A12-qF | ACCCTGGTGGACGATGTAGA | |
| A12-qR | GCTATGACCCGCTGATCGAA | |
| gyrB-qF | GATCCTCTCCAAGCGCATCC | |
| gyrB-qR | GCAGCACGGTCTTGTTCTTG | |
| pK18-A02L-up | TCGAGCTCGGTACCCATCCACCCACATTAGCTCCT | Mutant construction |
| pK18-A02L-down | TACGCAATGCGCCGGCCAGGGTTCTGCATTGATGA | |
| pK18-A02R-up | ATGCAGAACCCTGGACCGGCGCATTGCGTATAGAT | |
| pK18-A02R-down | CTCTAGAGGATCCCCGTACAACCCCGTGATGGCG | |
| A02-1st-R | CTGCACAAAGAGACCGCT | |
| A02-2nd-2-F | CATGCGAATGCGTACACG | |
| A02-2nd-2-R | GGTTCAGGCCAGGGAATG | |
| pK18-A12L-up | TCGAGCTCGGTACCCCGTTGGCCTCGTACAGC | |

| | |
|---|---|
| pK18-A12L-down | TCGACTACTCGGCGCGCAGGGCA |
| pK18-A12R-up | GCGCCGAGTAGTCGATCAGCTTGGGCGCG |
| pK18-A12R-down | CTCTAGAGGATCCCCAGCATGCCAATATCGGCA |
| A12-1st-R | GCGCAGGAGATCCTGC |
| A12-2nd-F | CGCCAGCTTGTAGGCC |
| A12-2nd-R | CGGTGCAGGACCTGATC |
| siRNA1_stem-loop | GTCGTATCCAGTGCAGGGTCCGAGGTATTCGCACTGGATACGACTGCCGCC |
| siRNA1-F | CTGAAGAATGAACTGGCGG |
| siRNA2_stem-loop | GTCGTATCCAGTGCAGGGTCCGAGGTATTCGCACTGGATACGACGGTTCCGC |
| siRNA2-F | ATCTTCGAGATGAAGCGGA |
| siRNA3_stem-loop | GTCGTATCCAGTGCAGGGTCCGAGGTATTCGCACTGGATACGACTTCATC |
| siRNA3-F | GTTCCCGGATCTTCGAGATGAA |
| siRNA4_stem-loop | GTCGTATCCAGTGCAGGGTCCGAGGTATTCGCACTGGATACGACAGTTCAA |
| siRNA4-F | GGAAACAGGCTGAGCTTGA |
| Univ_stem-loop-R | GTGCAGGGTCCGAGGT |
| Bv-xpsR-F | CGAAATCAGGAAGTGGCCCT |
| Bv-xpsR-R | GTCCCACCCCTCTGGACTAT |
| Bv-gyrB-F | GTGCATTTCATGGCCGATCC |
| Bv-gyrB-R | CGAACGCGAAATCGTCTTCC |

The lowercase sequence corresponds to the region that overlaps with the pTRV2 vector for cold fusion, while the uppercase indicates the primer sequence that binds to the target gene.
